# The topography of thought

**DOI:** 10.1093/pnasnexus/pgae163

**Published:** 2024-05-07

**Authors:** Jonah Berger, Olivier Toubia

**Affiliations:** Wharton School, University of Pennsylvania, Philadelphia, PA 19104, USA; Columbia Business School, Columbia University, New York, NY 10027, USA

**Keywords:** language, thought, natural language processing, automated textual analysis, academic success

## Abstract

Whether speaking, writing, or thinking, almost everything humans do involves language. But can the semantic structure behind how people express their ideas shed light on their future success? Natural language processing of over 40,000 college application essays finds that students whose writing covers more semantic ground, while moving more slowly (i.e. moving between more semantically similar ideas), end up doing better academically (i.e. have a higher college grade point average). These relationships hold controlling for dozens of other factors (e.g. SAT score, parents' education, and essay content), suggesting that essay topography encodes information that goes beyond family background. Overall, this work sheds light on how language reflects thought, demonstrates that how people express themselves can provide insight into their future success, and provides a systematic, scalable, and objective method for quantifying the topography of thought.

**Significance Statement**

Whether speaking, writing, or thinking, almost everything humans do involves language. But can the structure behind how people express their ideas shed light on their future success? Analyzing the language of over 40,000 college application essays finds that students whose writing covers more ground, and does so more coherently, perform better in college (i.e. end up having a higher grade point average (GPA)). This holds even controlling for dozens of other important predictors like SAT score, parents' education, or grades in high school. This work provides a systematic, scalable, and objective method for quantifying the topography of thought and illustrates how it might help explain people's future success.

## Introduction

Whether speaking, writing, or thinking, almost everything humans do involves language. But given language is a window into thought, does the way people express themselves shed light on their future success? In particular, does the semantic structure behind how people express their ideas relate to how well they end up doing academically?

Language reflects things about the people who produce it. Different people use words differently, so knowing what someone said or wrote provides insight into things like their personality and emotional state (1–5). Different types of function words (e.g. auxiliary verbs and conjunctions), for example, are associated with different cognitive styles (6), use of pronouns and negative emotion words is associated with lying (7), and use of pronouns and cognitive processing words can indicate an impending breakup (8).

But while it is clear that individual words reflect things about the people who use them, such words are almost always situated within a larger expression of ideas. Further, the ideas used to express thoughts, and the way people move between those ideas, can vary greatly. Someone writing about social issues could use more pronouns or emotional language, for example, but their writing could also cover more or less ground. They could connect a wide range of issues (e.g. climate change, racism, and sexism), or stay more circumspect, and focus on a narrower set (e.g. just climate change). Similarly, their arguments could slowly shift from one related idea to another, or they could move more quickly, jumping between ideas that are less clearly related.

Although existing work has focused on language's impact (i.e. how it influences readers, or listeners (9)), we suggest that such semantic features also reflect things about content producers (i.e. writers). Specifically, we hypothesize that the semantic ground people cover when expressing their ideas, and the speed with which they do so, provides insight into their future success.

A great deal of research suggests that associative abilities, or how people connect concepts to generate ideas, are linked to creativity and intelligence (10–14). Creative individuals are often good at combining unrelated concepts, for example, and connecting and integrating concepts in a meaningful way (13, 15).

Associative abilities are typically measured through tests. The Remote Associates Test (13), for example, gives people three words and asks them to think of a fourth word related to all three. Similarly, the Alternative Uses Test (16) has people think of as many uses as possible for a simple object like a brick.

But while tests can be useful, a more naturalistic means may also prove valuable (1). Almost 60 years ago, Koestler (17) theorized that one's thought process could be captured by plotting those thoughts in semantic space. Indeed, while it only examined individual words, recent work using latent semantic analysis (LSA) found that the forward flow of free thought predicts creativity (18).

Building on these notions, we predict that how people organize their ideas in a writing sample can shed light on their likely academic success years later.^a^ Research on discourse has used LSA to measure the relatedness, or semantic similarity, of chunks of texts (e.g. coherence (24, 25); see Ref. (26) for a review). More advanced textbooks, for example, tend to involve larger semantic jumps between adjoining portions of text (27). More recently, research has used advanced computational linguistic tools to explore whether the semantic progression of books, movies, and TV shows is linked to how popular they become (9).

Building on this work, we focus on two key dimensions. First, we consider the semantic *volume* of one's writing, or how much ground it covers (see Ref. (9) for related work on cultural success). French mathematician Poincaré (28) suggested that creating “consists of making new combinations of associative elements” and “the most fertile will often be those formed of elements drawn from domains which are far apart.” Indeed, covering more ground in one's writing (controlling for the length of what was expressed), involves generating ideas that combine more disparate concepts. Given such abilities have been linked to creativity, intelligence, and academic success (10, 12, 14, 15), we hypothesize that students whose essays cover more semantic ground will end up doing better in school.

Second, we consider how people *connect* ideas. More streamlined, logical thinkers should be able to find a parsimonious path through whatever space they want to cover, moving through ideas in a way that requires smaller cognitive jumps between adjoining concepts. Consistent with this notion, smaller semantic jumps between parts of discourse are taken to indicate more cohesive and comprehensible texts (see Ref. (26) for a review). Well-organized thought is also a key characteristic of cognitive functioning (29, 30). Consequently, we hypothesize that, controlling for the amount of ground covered, writing in a semantically slower, more cohesive manner, whereby each part of the text is semantically close to the adjoining parts, should also be linked to academic success (see Ref. (9) for related work on cultural success).

We test our predictions by analyzing the college application essays of over 20,000 students. Using a combination of natural language processing and machine learning, we test the link between semantic volume, speed, and future academic success. To increase confidence that the effects are not driven by ancillary factors that are correlated both with our focal measures and with student success, we include over 100 student-specific (e.g. SAT score, parents' education, and college major) and essay-specific (e.g. topics discussed and essay length) controls.

Along the way, our results also speak to the ongoing debate about the appropriateness of different information sources in college admission. While some schools are removing standardized tests from their admission criteria, due to the high correlation with socioeconomic status, some research suggests that certain features of application essays (i.e. topics and style) are equally or even more reflective of family background, and thus suffer from the same limitations (31). Consequently, we test whether the amount of ground covered and semantic speed are still related to student success even after controlling for factors that reflect family background.

## Natural language processing of over 40,000 college essays

### Method

We analyze college admissions essays and academic performance (i.e. GPA). A large public university required applicants to complete two admissions essays from a set of six prompts (e.g. describe a person or event that shaped your development). For 21,847 students (the total number available to us), we analyzed the relation between the text of both their essays and their cumulative GPA over the time they were enrolled at the school. Given privacy constraints, we were not able to see the raw texts of the essays, but we shared the relevant code with a member of the university, and they shared the resulting measures for each essay. The key features we extracted from the essays and used in our analyses are available at https://osf.io/aegx5/. To control for enrollment, we focus only on students who enrolled and matriculated.

Extant methods such as LSA (32), LDA (i.e. latent Dirichlet allocation (33)), or Doc2vec (34) allow representing documents as vectors in a latent semantic space. However, in order to test our predictions, we need to represent each document as an *ordered sequence of points* in the latent semantic space and develop measures that capture both the *local* properties of these points (e.g. the distance between each point and the next) as well as *global* properties of these points (e.g. the total ground covered by these points in the latent semantic space). To that end, using standard word embedding representations of each word as a starting point (we use Word2vec for simplicity and convenience, but other word embedding models could be used as well), we extract key writing features of interest by representing each essay as a progression of points in a latent semantic space (9). Similar to how a delivery driver's route can be broken up into multiple points, discourse (e.g. in this case essays) can be broken up into multiple points in a semantic trajectory. Essays (avg. length = 556.28 words) were broken into 25-word chunks,^b^ with each chunk embedded as a point using the standard Word2vec model (35). Each essay is represented by a path in the latent 300-dimensional semantic space, {*x_1_*,…,*x_T_*} where each point *x_t_* reflects the position of one chunk of text, and *T* is the number of points in the path.

**Fig. 1.**
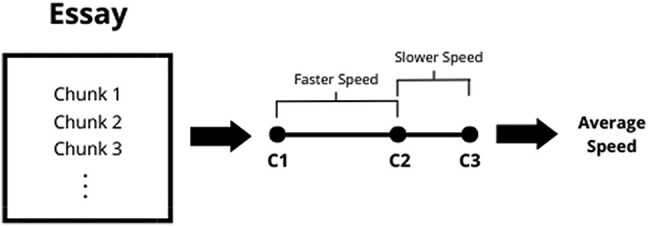
Illustration of speed.

Next, we use this representation to extract the local and global features of interest. To test our hypotheses, we measure the semantic speed with which essays move in this latent semantic space (a local feature). Note, this is not how fast the *reader* moves between chunks, but how quickly the *content* itself moves (i.e. the speed of semantic progression). Just as objects that cover a greater physical distance in the same amount of time can be described as moving faster, the same can be said of any discourse. Rather than dwelling on semantically related concepts, content that moves faster covers greater (semantic) distance in the same amount of time, jumping between content that is less semantically related (see Fig. 1).

To capture semantic speed, we measure the average semantic distance (or similarity) between essay chunks. Some individuals may be able to cover the same amount of semantic ground more quickly, effectively linking disparate ideas through more parsimonious semantic paths. Word embeddings capture semantic similarity (36–38), so consecutive chunks that are further away discuss content that is more semantically different (9). To capture this, we measure the average Euclidian distance between consecutive points in each essay. We take the average of distance(*t*) from *t* = 1 to *T*−1, where distance(*t*) is the Euclidean distance between *x_t_* and *x_t +_*  _1_. Speed is then simply defined as the total distance divided by *T*−1. See Supplementary material for a validation study.

**Fig. 2.**
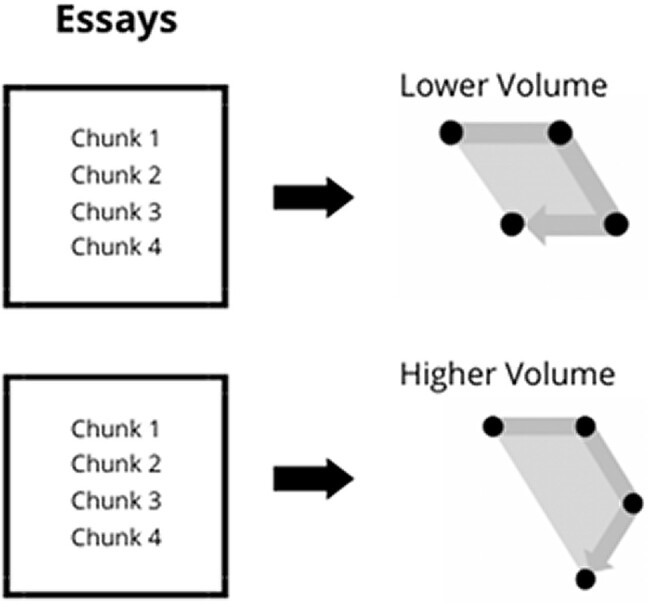
Illustration of volume.

As discussed, we also measure the amount of semantic ground covered by an essay. Unlike speed, which is about the average semantic distance between *consecutive* pieces of content, the ground covered depends on the set of content as a whole, i.e. it is a global feature. Regardless of their sequence, it examines whether the set of points cover a smaller part of semantic space, or a larger one (see Fig. 2 for an illustration, where volume is varied while holding speed constant).

To measure this, we identify the minimum volume ellipsoid containing all of the points in each essay. That is, we solve an optimization problem to find the ellipsoid that cover all the points {*x_1_*, …*x_T_*} with the minimal volume (see Supplementary material for more detail). We normalize volume to account for the number of points *T*. Controlling for essay length, covering more ground, or greater volume requires combining concepts that are less semantically related, and this measure is reliably linked to human perceptions of how much ground content covers (9).

To account for the skewness of their distributions, we log transform these features and standardize them for ease of interpretation. Results are the same if the variables are not log-transformed. We standardize all other variables for which we report coefficients. To examine the link between these features and subsequent grades, we average essay features for the two essays written by each student and use the student as the unit of analysis.^c^ See Supplementary material for a correlation matrix of the main variables and an analysis of multicollinearity (including variance inflation factor). Using ridge regression to mitigate any potential effects of multicollinearity leads to the same results (see Supplementary material).

### Results

Consistent with our predictions, both speed and volume were linked to success. An initial ordinary least squares regression with speed and volume as independent variables finds that students whose writing covered more ground ended up doing better academically (i.e. higher GPA, *β* = 0.165, *P* < 0.01, *t* = 12.70), as did students who covered this ground moving more slowly (*β* = −0.045, *P* < 0.01, *t* = −3.37).^d^

That said, while these results are intriguing, one could argue they are driven by a number of other factors unrelated to the topography of thought. Consequently, we include a variety of student- and essay-specific controls to test robustness.

#### Student-specific controls

First, students with stronger past academic performance likely get better grades, and may also write differently, so we control for this in a number of ways, including SAT scores (both Math and Verbal) as well as high school rank (i.e. the student's relative rank in their high school). This also ensures that we control for the information SAT scores encode about the student's family background and socioeconomic status.

Second, we further control for family background using mother and father's education (using fixed effects for the highest level of education reached by each parent). This controls for the fact that students whose parents were more successful at school may be more likely to be successful themselves and to write differently.

Third, gender and ethnicity may also shape writing and academic achievement, so we control for these factors as well (again using fixed effects).

Fourth, students applying to different majors may write differently and get different grades, so we control for the college where they enrolled (e.g. engineering or liberal arts).

Fifth, we include other factors that might impact or relate to writing or academic achievement like whether students came from instate or were automatically admitted.

#### Essay-specific controls

Beyond fixed aspects of each student, aspects of their writing may also play a role, so we controlled for those as well. First, the specific topics students write about may be related to their academic achievement, and/or shape the volume or speed of their thought expression, so we control for the topics of each essay using LDA (33). We use 100 topics and control for the prevalence of each topic in each essay. This also allows us to control for the information about family background that can be encoded in essay content (31).

Second, prior work indicates that using more categorical or dynamic language relates to academic achievement (6), so we control for that using what is now labeled analytic thinking in Linguistic Inquiry and Word Count (39). This again controls for the fact that essay style reflects family background (31).

Third, although volume and speed are already normalized by the number of chunks of text in the essay, we further control for essay length in three ways (i.e. number of words, number of sentences, and number of chunks of text, all log-transformed).

Fourth, the essay prompt picked by students likely impacts what they write about, and could also be correlated with academic achievement, so we include fixed effects for each prompt.

#### Results with controls

Even including these over 100 controls, though, results remain the same (Table 1, Model 1). Students whose writing covered more ground performed better in college (i.e. higher GPA, *β* = 0.046, *P* < 0.01, *t* = 3.73) as did students whose writing moved more slowly in the latent semantic space while doing so (*β* = −0.024, *P* < 0.05, *t* = −2.09).

**Table 1.** Topography of thought and academic performance.

	Model 1	Model 2
Focal variables		
Volume	0.046^a^	0.093^a^
Speed	−0.024^b^	**—**
** **Min. required speed	**—**	−0.073^a^
** **Circuitousness	**—**	0.007
Student-specific controls		
** **SAT Math	0.177^a^	0.177^a^
** **SAT Verbal	0.102^a^	0.103^a^
** **High school rank	0.145^a^	0.144^a^
** **Father's education	Yes	Yes
** **Mother's education	Yes	Yes
** **Gender	Yes	Yes
** **Ethnicity	Yes	Yes
** **College within univ.	Yes	Yes
** **Year of application	Yes	Yes
** **TX High School dummy	Yes	Yes
** **Auto-admit dummy	Yes	Yes
Essay-specific controls		
** **LDA topic weights	Yes	Yes
** **Analytic thinking	0.026^a^	0.027^a^
** **Log (# of words)	0.079^a^	0.080^a^
** **Log (# of sentences)	0.009	0.016
** **Log (# of chunks)	−0.012	−0.038
** **Essay prompt	Yes	Yes
Number of parameters	145	146
Number of observations	21,847	21,847
*R* ^2^	0.325	0.326
Adjusted *R*^2^	0.321	0.321

All variables for which coefficients are reported are standardized.

^a^ *P* < 0.01. ^b^*P* < 0.05.

While recent work notes that the content and style of college application essays can encode information about family background (31), the fact that volume and speed are related to performance even after controlling for multiple factors reflecting family background (e.g. parents' education and the student's SAT scores), as well as essay content (using topic modeling) and style (using LIWC), indicates that volume and speed capture something beyond just family background.

While speed and volume are statistically significant in our regression, one might wonder how much they improve the ability to explain student success. As a benchmark, Pennebaker et al. (6) report how much the adjusted *R*^2^ is increased when CDI (what is now described as analytic thinking) is added to a simple forced-entry linear regression with SAT scores and high school rank as the only explanatory variables. In our case, a simple forced-entry linear regression on average GPA found that SAT Math, SAT Verbal and high school rank yield an adjusted *R*^2^ of 0.214.^e^ Adding the simple CDI Index from function word analyses of the admissions essays increases the adjusted *R*^2^ to 0.225. In comparison, adding speed and volume (instead of CDI) increases the adjusted *R*^2^ to 0.223. Thus, it appears that speed and volume have similar explanatory power as CDI. Full regressions with 145 and 146 covariates have an adjusted *R*^2^ of 0.321. Removing any of these variables from these regressions has a negligible impact on adjusted *R*^2^.

#### Robustness

Rather than reflecting something about the students, one could wonder if the results are driven by how essays are graded, or more generally if the features identified here are merely characteristic of well-composted texts. If students write essays for class the same way they write their application essays, for example, and class essays that cover more ground and do so more cohesively get higher grades, then maybe that is the reason that students who write this way have a higher GPA. This would be particularly concerning if this were driven by writing norms passed on through channels that reflect the student's family background.

To test this possibility, we examine just the colleges at the university that do not require intensive essay writing (i.e. engineering, natural sciences, nursing, and geology). In these colleges, grades are often based on factors others than just how students write, and essays are often not a major component of the grading process. Results, however, remain the same (*β*_Volume_ = 0.050, *P* < 0.01, *t* = 2.77; *β*_Speed_ = −0.031, *P* = 0.07, *t* = −1.80), casting doubt on the possibility that the observed relationship is driven by how essays are graded.

Alternatively, one could wonder if the results are somehow driven by essay prompts that ask students to reflect on their personal experiences. Maybe students who describe more diverse and rich personal experiences cover more volume in their essays, but it is the diversity of experiences (another potential reflection of family background) that then drive their academic success, rather than what their writing reflects about how they think.

To test this possibility, we examine just the essay prompts that do not ask students to reflect on past experiences (i.e. asking them to “describe a potential classmate that you believe you could learn from” instead). Results, however, remain the same (*β*_Volume_ = 0.039, *P* < 0.01, *t* = 4.06; *β*_Speed_ = −0.022, *P* < 0.05, *t* = −2.53), casting doubt on the possibility that the observed relationship is simply driven by some people having richer and more diverse experiences to write about.

#### What speed indicates

While the relationship with speed is intriguing, one could wonder whether it is driven by which concepts were *selected*, or how they were *organized*. A delivery company could do their job with a more cohesive route (i.e. less distance traveled), for example, either because they *select* stops that are not too far apart, or because given the required stops, they *organize* each route in a way that minimizes the driving distance. Similarly, speed's negative effect could imply that high-performing students select and combine concepts that do not require as long a path to connect (controlling for the amount of ground covered), or that given the concepts discussed, high-performing students organize them in a more optimal sequence that requires a shorter path.

To tease these aspects apart, we decompose speed into two components: (i) the minimum required speed to cover the content and (ii) circuitousness, or how optimally the text is organized given its content. We solve for the shortest path, and hence the minimum required speed, by solving a version of the classic Traveling Salesperson optimization problem (40). Circuitousness is measured by the extent to which the actual latent semantic path differs from the shortest path that starts and ends at the same point, and visits all the same points in between (9). Formally, circuitousness is given by: circuitousness = speed/(minimum required speed). Given that all variables are log-transformed, log(speed) = log(circuitousness) + log(minimum required speed).

Results (Table 1, Model 2) indicate that speed's effect is driven by the minimum speed required (*β* = −0.073, *P* < 0.01, *t* = −4.79) and not circuitousness (*β* = 0.007, *P* = 0.32, *t* = 1.01). Combined with the effect of volume, this suggests the link between writing and academic achievement is driven more by the selection of concepts rather than the specific sequence in which the selected concepts are ordered. Rather than just *structuring* their essays more efficiently, students who perform better seem to *select* concepts that allow them to explain ideas with less semantic distance.

## General discussion

Scientists have long theorized about the topography of thought. Albert Einstein famously wrote that “combinatory play seems to be the essential feature in productive thought” (41) and interviews with over 30 Nobel laureates concluded that integration, where “multiple separate elements retain their discreteness and identity while connected and operating together in a whole” is the characteristic result of the cognitive creative process ((42), p. 9).

But while great thinkers may combine thoughts and concepts in novel and important ways, how to actually measure such semantic organization is less clear.

By integrating theories from cognitive psychology and creativity with natural language processing and computational linguistic tools, this paper begins to quantify the connection between the topography of thought and academic outcomes. In particular, results suggest that people who cover more semantic ground when expressing their ideas, and select concepts that allow them to cover such ground more cohesively (i.e. with smaller semantic jumps), end up performing better academically.

Although application essays have no direct impact on students' performance in college once enrolled, the way a student expresses ideas in these essays, when quantified properly, can shed light on their future success. This suggests that the topography of someone's written output provides a window into their thought process which can be systematically and objectively quantified for novel information and insight.

Given the correlation between standardized tests and socioeconomic background (43), many schools are decreasing their reliance on these measures and putting more weight on things like application essays. But as recent work has shown (31), essay's style and topical content suffer from the similar limitations as standardized scores (i.e. correlation with family background). That said, our results demonstrate that essay topography encodes information that is predictive of academic success while not being a direct reflection of family background.

Based on the literature reviewed, we have argued that people whose writing covers more semantic ground should be more creative. Indeed, ancillary data we analyzed is consistent with this notion (see Supplementary material). People were asked to generate ideas for a new health-related smartphone app, and, consistent with our theorizing, ideas that covered more ground (while moving semantically slower) were seen as more creative (see Supplementary material). Such ability to generate creative ideas, in turn, should drive academic success (10, 14). To the extent that associative abilities have been shown to be linked to general intelligence (10–14), covering more ground, and doing so while moving semantically slower, may also be linked to intelligence more generally.

### Limitations and directions for future research

This work is not without limitations. College essays are written for a particular audience, and may be shaped by parents, teachers, and even professional counselors. Consequently, one could wonder whether rather than reflecting how students think, speed and volume simply reflect how involved their family is in the process or how much help the student received. Maybe wealthier students' essays were shaped by admissions consultants, for example. While this is certainly possible, outside essay help, by itself, is unlikely to explain the results. Such students would have to had help both in the application process itself, and enough during college to shape their GPA. Further, given our analysis focused on a public university, it seems less likely that a large number of students received such consistent help, and we already control for family background in a number of ways. The ancillary data also suggest that speed and volume are linked to creativity even outside a context where others could be involved in content generation. That said, future work could examine this point in greater detail.

While we analyzed essays from tens of thousands of students from a large public university, one could also wonder whether the results generalize to other settings. This university attracts students from a range of racial, cultural, and economic backgrounds, and Caucasian students make up less than 55% of the student body, but future work could examine whether these results extend outside of the United States. Although the ability to connect disparate ideas may be linked to creativity across cultures, given cultural differences in tendencies (and values) for holistic versus analytic thinking (see Ref. (44) for a review), this may be a fruitful area for further consideration.

Our findings also raise other interesting questions for future work. First, might similar approaches help explain success in other domains? Might the content of cover letters help explain job performance, for example, or might the structure of an academic's early papers help predict later success?

Second, are these features truly linked to creativity, as suggested? Work on creativity often distinguishes between divergent thinking and convergent thinking processes, which respectively consist in generating a broad range of ideas related to a given stimulus and discerning which ideas are most appropriate (12, 45). Future work could study the relationship between convergent and divergent thinking on the one hand and semantic speed and volume on the other. Such work might enable researchers to detect convergent and divergent thinking in naturalistic texts and improve our understanding of the link between these constructs and success in creative domains.

Alternatively, work might examine how our results link to depth of understanding. Covering more ground cohesively when expressing ideas, for example, may reflect deeper understanding of a topic. The ability to slowly and stepwise traverse a series of concepts in a coherent way may thus indicate greater insight about a particular topic.

Third, which types of content might be more diagnostic of success and why? Compared to writing, for example, speaking often involves less deliberation (46, 47). But while this may lead spoken content to better reflect one's natural thought process, whether this better predicts life outcomes may depend on whether the context predicted is more reflexive or deliberative.

Fourth, given the advent of Generative AI, it might be interesting to explore whether algorithms can be set up to artificially generate texts with various configurations of semantic speed and volume. Such exercise might improve our ability to identify a causal link between the topography of thought and success and provide the foundations for tools that would generate “optimal” texts in various contexts.

More broadly, these findings highlight how natural language processing can shed light on a range of interesting questions (48, 49). The digitization of everything from interpersonal communication and college applications to counseling conversations and online posts has provided a wealth of information about people, relationships, and society more generally. But extracting insights from this data requires the right tools. Advances in computer science, computational linguistics, and other areas have provided a range of new, exciting approaches. By leveraging these approaches, hopefully we can extract more wisdom from words.

## Supplementary Material

pgae163_Supplementary_Data

## Data Availability

Given privacy constraints, the authors were not able to see the raw texts of the essays, but they shared the relevant code with a member of the university, and they shared the resulting measures for each essay. The key features they extracted from the essays and used in their analyses are available at https://osf.io/aegx5/.

## Notes

a There is a great deal of work on automated essay scoring (see Refs. (19, 20) for recent reviews), some of which showing moderate correlations with things like GPA. That said, this paper expands on that work in some important ways. First, most automated essay scoring work has focused on measuring writing skills (e.g. essay quality or grammatical errors), rather than extracting features that might reflect how students think. Second, while simple features like word and sentence length have been helpful to measuring things like readability (e.g. Flesch–Kincaid grade level), more “challenging discourse-level problems that involve the computational modeling of different facets of text structure, such as coherence, thesis clarity, and persuasiveness…require an understanding of essay content, which is largely beyond the reach of state-of-the-art essay scoring engines” ((19), p. 6,300). As such, work linking automated essay features and GPA (e.g. (21–23)) does not include more complex features such as the ones we consider here. Consequently, this paper expands the features examined, links them to important student outcomes, and suggests how they might reflect patterns of thought.

b We do not break up sentences, and hence some chunks may have more 25 words.

c Prior work in similar data, Pennebaker et al. (6) found categorical-dynamic index (CDI) showed a reliability of *r* = 0.38 across essays. The reliability of volume is similar (*r* = 0.35), while the reliability of speed is slightly smaller (*r* = 0.26).

d These results highlight the importance of not just considering local measures like speed that compare adjacent pieces of content, but also global ones such as volume that jointly capture all content pieces. While speed’s relationship with GPA is positive when considered in isolation (*r* = 0.118, *P* < 0.01), content which moves faster also tends cover more ground (correlation between volume and speed = 0.893, *P* < 0.001). Speed’s relationship with GPA becomes negative once volume is accounted for, however, indicating that rather than making large semantic jumps, it is actually best to cover a lot of semantic ground but do so with smaller semantic jumps. Better basketball players may tend to weigh more, for example, but this is because height and weight are correlated. Once height is controlled for, weight may actually have a negative effect.

e Our numbers are similar but not directly comparable to Pennebaker et al.’s (6), because our dependent variable is the average GPA, and we control separately for SAT Math and SAT Verbal.

## References

[1] Arnulf JK, Larsen KR, Martinsen OL, Nimon KF. 2021. Semantic algorithms in the assessment of attitudes and personality. Front Psychol. 12:720559. 34367039 10.3389/fpsyg.2021.720559 PMC8342853

[2] Boyd RL, Schwartz HA. 2021. Natural language analysis and the psychology of verbal behavior: the past, present, and future states of the field. J Lang Soc Psychol. 40(1):21–41. 34413563 10.1177/0261927x20967028 PMC8373026

[3] Pennebaker JW, Mehl MR, Niederhoffer KG. 2003. Psychological aspects of natural language use: our words, our selves. Annu Rev Psychol. 54(1):547–577. 12185209 10.1146/annurev.psych.54.101601.145041

[4] Park G, et al 2015. Automatic personality assessment through social media language. J Pers Soc Psychol. 108(6):934–952. 25365036 10.1037/pspp0000020

[5] Sap M, et al 2014. Developing age and gender predictive lexica over social media. In: *Proceedings of the 2014 Conference on Empirical Methods in Natural Language Processing (EMNLP)*; October 25–29, 2014; Doha, Qatar. p. 1146–1151.

[6] Pennebaker JW, Chung CK, Frazee J, Lavergne GM, Beaver DI. 2014. When small words foretell academic success: the case of college admissions essays. PLoS One. 9(12):e115844. 25551217 10.1371/journal.pone.0115844 PMC4281205

[7] Newman ML, Pennebaker JW, Berry DS, Richards JM. 2003. Lying words: predicting deception from linguistic styles. Pers Soc Psychol Bull. 29(5):665–675. 15272998 10.1177/0146167203029005010

[8] Seraj S, Blackburn KG, Pennebaker JW. 2021. Language left behind on social media exposes the emotional and cognitive costs of a romantic breakup. Proc Natl Acad Sci U S A. 118(7):e2017154118. 10.1073/pnas.2017154118 PMC7896325 33526594

[9] Toubia O, Berger J, Eliashberg J. 2021. How quantifying the shape of stories predicts their success. Proc Natl Acad Sci U S A. 118(26):e2011695118. 10.1073/pnas.2011695118 PMC8256009 34172568

[10] Barron F, Harrington DM. 1981. Creativity, intelligence, and personality. Annu Rev Psychol. 32(1):439–476.

[11] Lee CS, Huggins AC, Therriault DJ. 2014. A measure of creativity or intelligence? Examining internal and external structure validity evidence of the Remote Associates Test. Psychol Aesthet Creat Arts. 8(4):446–460.

[12] Lee CS, Therriault DJ. 2013. The cognitive underpinnings of creative thought: a latent variable analysis exploring the roles of intelligence and working memory in three creative thinking processes. Intelligence. 41(5):306–320.

[13] Mednick S . 1962. The associative basis of the creative process. Psychol Rev. 69(3):220–232. 14472013 10.1037/h0048850

[14] Plucker JA . 1999. Is the proof in the pudding? Reanalyses of Torrance's (1958 to present) longitudinal data. Creat Res J. 12(2):103–114.

[15] Benedek M, Könen T, Neubauer AC. 2012. Associative abilities underlying creativity. Psychol Aesthet Creat Arts. 6(3):273–281.

[16] Guilford JP, Merrifield PR, Wilson RC. 1958. Unusual uses test. Orange (CA): Sheridan Psychological Services.

[17] Koestler A . 1964. The act of creation: a study of the conscious and unconscious processes of humor, scientific discovery and art. New York (NY): Dell Books.

[18] Gray K, et al 2019. “Forward flow”: a new measure to quantify free thought and predict creativity. Am Psychol. 74(5):539–554. 30667235 10.1037/amp0000391

[19] Ke Z, Ng V. 2019. Automated essay scoring: a survey of the state of the art. In: *Proceedings of the Twenty-eighth International Joint Conference on Artificial Intelligence*. p. 6300–6308.

[20] Ramesh D, Sanampudi SK. 2022. An automated essay scoring systems: a systematic literature review. Artif Intell Rev. 55:2495–2527. 34584325 10.1007/s10462-021-10068-2 PMC8460059

[21] Powers DE, Burstein JC, Chodorow M, Fowles ME, Kukich K. 2000. *Comparing the validity of automated and human essay scoring*. GRE Board Research Report No. 98-08aR. Princeton (NJ): Educational Testing Service.

[22] Ramineni C . 2013. Validating automated essay scoring for online writing placement. Assess Writ. 18(1):40–61.

[23] Attali Y, Bridgeman B, Trapani C. 2010. Performance of a generic approach in automated essay scoring. J Technol Learn Assess. 10(3). http://www.jtla.org.

[24] Somasundaran S, Burstein J, Chodorow M. 2014. Lexical chaining for measuring discourse coherence quality in test-taker essays. In: *Proceedings of COLING 2014, the 25th International Conference on Computational Linguistics: Technical Papers*; August 2014; Dublin, Ireland. p. 950–961.

[25] Tay Y, Phan MC, Tuan L, Hui S. 2018. SkipFlow: incorporating neural coherence features for end-to-end automatic text scoring. In: *The Thirty-Second AAAI Conference on Artificial Intelligence (AAAI-18)*. p. 5948–5955.

[26] Foltz PW . 2007. Discourse coherence and LSA. In: Landauer TK, McNamara DS, Dennis S, Kintsch W, editors. Handbook of latent semantic analysis. Mahwah (NJ): Lawrence Erlbaum Associates Publishers. p. 179–196.

[27] Foltz PW, Kintsch W, Landauer TK. 1998. The measurement of textual coherence with latent semantic analysis. Discourse Process. 25(2–3):285–307.

[28] Poincaré H . 1913. The foundations of science. New York: The Science Press.

[29] Elvevåg B, Foltz PW, Weinberger DR, Goldberg TE. 2007. Quantifying incoherence in speech: an automated methodology and novel application to schizophrenia. Schizophr Res. 93(1–3):304–316. 17433866 10.1016/j.schres.2007.03.001 PMC1995127

[30] Elvevåg B, et al 2017. Thoughts about disordered thinking: measuring and quantifying the laws of order and disorder. Schizophr Bull. 43(3):509–513. 28402507 10.1093/schbul/sbx040 PMC5464160

[31] Alvero AJ, et al 2021. Essay content and style are strongly related to household income and SAT scores: evidence from 60,000 undergraduate applications. Sci Adv. 7(42):eabi9031. 34644119 10.1126/sciadv.abi9031 PMC8514086

[32] Deerwester S, Dumais ST, Furnas GW, Landauer TK, Harshman R. 1990. Indexing by latent semantic analysis. J Am Soc Inform Sci. 41:391–407.

[33] Blei DM, Ng AY, Jordan MI. 2003. Latent Dirichlet allocation. J Mach Learn Res. 3(2):993–1022.

[34] Le Q, Mikolov T. 2014. Distributed representations of sentences and documents. In: *Proceedings of the 31st International Conference on Machine Learning*. 32(2):1188–1196.

[35] Mikolov T, Grave É, Bojanowski P, Puhrsch C, Joulin A. (2018). Advances in pre-training distributed word representations. In: *Proceedings of the Eleventh International Conference on Language Resources and Evaluation (LREC 2018)*; May 2018; Miyazaki, Japan.

[36] Bhatia S . 2017. Associative judgment and vector space semantics. Psychol Rev. 124(1):1–20. 28004958 10.1037/rev0000047

[37] Garg N, Schiebinger L, Jurafsky D, Zou J. 2018. Word embeddings quantify 100 years of gender and ethnic stereotypes. Proc Natl Acad Sci U S A. 115(16):3635–3644. 10.1073/pnas.1720347115 PMC5910851 29615513

[38] Kozlowski AC, Taddy M, Evans JA. 2019. The geometry of culture: analyzing the meanings of class through word embeddings. Am Sociol Rev. 84(5):905–949.

[39] Pennebaker JW, Boyd RL, Jordan K, Blackburn K. 2015. The development and psychometric properties of LIWC2015. Austin (TX): University of Texas at Austin.

[40] Dantzig GB . 1998. Linear programming and extensions. vol. 48. Princeton (NJ): Princeton University Press.

[41] Hadamard J . 1945. An essay on the psychology of invention in the mathematical field. New York (NY): Dover Publications.

[42] Rothenberg A . 2014. Flight from wonder: an investigation of scientific creativity. New York (NY): Oxford University Press.

[43] Dixon-Román EJ, Everson HT, McArdle JJ. 2013. Race, poverty and SAT scores: modeling the influences of family income on black and white high school students’ SAT performance. Teach Coll Rec. 115(4):1–33.

[44] Nisbett RE, Peng K, Choi I, Norenzayan A. 2001. Culture and systems of thought: holistic versus analytic cognition. Psychol Rev. 108(2):291–310. 11381831 10.1037/0033-295x.108.2.291

[45] Goldschmidt G . 2016. Linkographic evidence for concurrent divergent and convergent thinking in creative design. Creat Res J. 28(2):115–122.

[46] Ochs E . 1979. Planned and unplanned discourse. In: Givon T, editor. Discourse and syntax. New York (NY): Academic Press. p. 51–80.

[47] Oba D, Berger J. 2024. How communication mediums shape the message. J Consum Psychol. 10.1002/jcpy.1372.

[48] Berger J, Packard G. 2022. Using natural language processing to understand people and culture. Am Psychol. 77(4):525–537. 34914405 10.1037/amp0000882

[49] Jackson JC, et al 2022. From text to thought: how analyzing language can advance psychological science. Perspect Psychol Sci. 17(3):805–826. 34606730 10.1177/17456916211004899 PMC9069665

