# Supplementary material for: The topography of thought
Source: PNAS Nexus. 2024 May 7;3(5):pgae163. doi: 10.1093/pnasnexus/pgae163 (PMC11075530; doi:10.1093/pnasnexus/pgae163)
Supplement: pgae163_Supplementary_Data [file pgae163_supplementary_data.docx]

**Supplemental Materials**

**Volume Computation**

We find the minimum-volume enclosing ellipsoid containing points{*x_1_*,…*x_T_*}, by solving the following optimization problem:

Maximize*_d_*_,_*_A_* det(*A*)

subject to:

(*x_t_ -d*)^T^>*A*(*x_t_* -*d*)≤1, *t* =1, … , *T*

*A* is a positive definite matrix.

See Toubia, et al., (2021) for details on solving this problem, depending on the rank of {*x_1_*,…*x_T_*}. The lengths of the axes of the ellipsoid are given by the inverse of the square root of the eigenvalues of the (positive definite) matrix that defines this ellipsoid. The product of the length of the axes equals the volume of the ellipsoid relative to a unit sphere. We normalize this measure by using the geometric mean (rather than the product) of the lengths of the axes of the minimum-volume ellipsoid as our normalized measure of volume.

**Validation of Speed**

As noted in the text, prior work demonstrates that our speed and volume measures accurately capture those constructs, but we conduct several additional validation tests in the academic essay domain. To do so, we collect a sample of 216 publicly available college application essays from a variety of sources (e.g., https://www.thecrimson.com/topic/sponsored-successful-harvard-essays-2022/, <https://www.collegeessayguy.com/blog/college-essay-examples#Personal%20Statement%20Examples>).

*Automated Validation of Speed*. First, we randomly draw 10,000 target chunks (without replacement) across all essays that have at least 10 chunks. Then, for each target chunk, we randomly select four comparison chunks: (1) an adjacent chunk, (2) a chunk located within +/- 5 positions of the initial chunk, (3) a chunk from anywhere in the same essay, and (4) a chunk from another essay. Finally, we compute the word embedding distance between the target chunk and each of these four types of chunks, averaging the distances for each type across all 10,000 target chunks.

Consistent with the notion that our automated measure of speed captures semantic distance, results indicate that adjacent chunks of text from the same essay (M = 0.409) are semantically closer (on average) than nearby chunks from the same essay (M = 0.421), which are closer than chunks from anywhere within the same essay (M = 0.427), which are closer than chunks from different essays (M = 0.456). All pairwise comparisons are significant at p < 0.01.

*Human Perceptions of Speed*. We also further test whether our speed measure captures perceived similarity. We randomly pick 50 essays with at least 10 chunks, and randomly select one chunk A from each. Then, for each of these chunks, we construct two triplets. First, we compute the distance between the target chunk and all other chunks in the same essay, and randomly pick one chunk from the bottom and one from the top quartile (i.e., one chunk that is close to the target chunk and one that is far). We randomly label these two chunks B_same_ and C_same_. Second, we randomly draw (without replacement) two other essays (among the set of 50), randomly pick one chunk in the bottom quartile of distances (to the target chunk) for one essay and one chunk in the top quartile of distances for the other essay. Together, this gives us 100 triplets: {A, B_same_,C_same_} and {A, B_other_,C_other_} for each A.

For each of the 100 triplets, hypothesis-blind research assistants (N = 4) coded whether chunk B or chunk C was more similar to chunk A (i.e., “the content focuses on similar topics, themes, or ideas”). Majority rule determined which chunk was closer to A (in the 15% of cases that were ties, they were broken randomly). For each triplet, the position of each chunk in the word embedding space also determined which chunk was closer to A.Consistent with the notion that the automated measure is accurately capturing human perceptions of similarity and speed, it agreed with human judgments 71.5% of the time. Further, its accuracy was similar to the probability of agreement between the two human judges (69%) indicating that the automated measure is reasonably consistent with human perceptions and similarly reliable.

**Multicollinearity**

We explore multicollinearity, and in particular the correlation between the focal variables (speed and volume) and the control variables. We address this several ways.

First, we report the correlation matrix between GPA, focal variables, and some of the key control variables. As shown in Table S1, the focal variables are not highly correlated with HS rank, SAT scores or CDI. There is a high correlation between speed, minimum required speed, and volume. This is why we include both volume and speed, or both volume and minimum required speed, in our regressions. Indeed, including only speed or volume in the regression could lead to incorrect parameter estimates, due to the high correlation between the included variable and the omitted variable. When both variables are included, as in our case, the fact that they are highly correlated widens the confidence intervals of the parameter estimates, making it harder to find significant effects (Allen, 1997).

Table S1. Correlation Between Key Variables

|  | HS rank | SAT Math | SAT Verbal | CDI | log(speed) | log(circuitousness) | log(min. required distance) | log(volume) |
| --- | --- | --- | --- | --- | --- | --- | --- | --- |
| SAT Math | -0.01 |  |  |  |  |  |  |  |
| SAT Verbal | 0.00 | 0.59 |  |  |  |  |  |  |
| CDI | -0.04 | 0.20 | 0.22 |  |  |  |  |  |
| log(speed) | -0.04 | 0.09 | 0.13 | 0.17 |  |  |  |  |
| log(circuitousness) | -0.02 | 0.01 | 0.01 | -0.15 | 0.26 |  |  |  |
| log(min. required distance) | -0.03 | 0.09 | 0.12 | 0.24 | 0.90 | -0.20 |  |  |
| log(volume) | -0.04 | 0.12 | 0.15 | 0.26 | 0.89 | -0.03 | 0.92 |  |
| GPA | 0.22 | 0.35 | 0.37 | 0.19 | 0.12 | 0.05 | 0.10 | 0.14 |

Next, we examine the correlation between our measures of average speed, normalized volume and circuitousness and all independent variables. Given there are 147 variables in the analysis, it is impractical to report the full correlation matrix. Instead, we report the correlation between the four topography variables and each group of variables (i.e., fixed effects for father’s education, mother’s education, etc.). For groups with a single variable (e.g., SAT math), we simply report the correlation. For groups with multiple variables (e.g., father’s education), we report the average and max absolute value of the correlations. Results (Table S2) indicate that the correlation between any of the topography variables and the others is always below the standard threshold of 0.7 (Dormann et al., 2013).

Table S2. Correlation Between Topography Variables and All Variables / Groups of Variables

|  | Volume | Speed | Min required speed | Circuitousness |
| --- | --- | --- | --- | --- |
| ***Student-specific controls*** |  |  |  |  |
| SAT Math | 0.12 | 0.09 | 0.09 | 0.01 |
| SAT Verbal | 0.15 | 0.13 | 0.12 | 0.01 |
| High school rank | -0.04 | -0.04 | -0.03 | -0.02 |
| Father’s education: average/max absolute value | 0.06/0.11 | 0.05/0.10 | 0.05/0.09 | 0.01/0.02 |
| Mother’s education: average/max absolute value | 0.06/0.07 | 0.05/0.09 | 0.05/0.06 | 0.01/0.01 |
| Gender=female | 0.09 | 0.09 | 0.08 | 0.04 |
| Ethnicity: average/max absolute value | 0.03/0.09 | 0.02/0.07 | 0.02/0.07 | 0.01/0.03 |
| College within Univ.: average/max absolute value | 0.01/0.02 | 0.01/0.03 | 0.01/0.02 | 0.01/0.03 |
| Year of Application:  average/max absolute value | 0.01/0.02 | 0.01/0.02 | 0.01/0.02 | 0.03/0.05 |
| TX HS dummy | -0.01 | -0.01 | -0.02 | 0.02 |
| Auto-admit dummy | -0.06 | -0.05 | -0.04 | -0.02 |
| ***Essay-specific controls*** |  |  |  |  |
| LDA topic weights: average/max absolute value | 0.07/0.29 | 0.06/0.26 | 0.06/0.26 | 0.03/0.10 |
| CDI | 0.26 | 0.17 | 0.24 | -0.15 |
| Log(# of words) | 0.05 | 0.09 | -0.16 | 0.54 |
| Log(# of sentences) | 0.30 | 0.33 | 0.11 | 0.50 |
| Log(# of chunks) | 0.17 | 0.21 | -0.04 | 0.56 |
| Essay prompt: average/max absolute value | 0.01/0.02 | 0.01/0.02 | 0.01/0.01 | <0.01/0.01 |

We also compute the Variance Inflation Factors (VIF) for volume, speed, min. required speed and circuitousness. We first compute the VIF for each variable when it is entered by itself with the 143 other regressors. As shown in the first column of Table S3, the VIF is always far below 10, which is the standard cutoff (Dormann et al., 2013).

Next, we compute the VIF for the topography variables when they are entered together, as in the two models reported in Table 1. In Model 1, volume and speed are entered together, and the VIFs remain below 10. In Model 2, volume, min. required speed and circuitousness are entered together, and the VIFs for volume and min. required speed are both around the cutoff of 10. This reflects the high correlation between these two variables, discussed above.

Table S3. Variance Inflation Factors (VIF)

|  | VIF if entered without other topography variables | VIF in Model 1 | VIF in Model 2 |
| --- | --- | --- | --- |
| Volume | 2.45 | 6.39 | 10.46 |
| Speed | 2.03 | 5.29 | --- |
| Min. required speed | 2.23 | --- | 9.71 |
| Circuitousness | 1.55 | --- | 1.62 |
| Num parameters | 144 | 145 | 146 |

Finally, one standard way to alleviate potential effects of multicollinearity is to use ridge regression (Allen, 1997). Recall that ridge regression replaces the OLS estimate (X’X)^-1^X’y with (X’X+*k*I)^-1^X’y. We standardize each column of the matrix X, and vary *k* from to 0 .1 to 1 in 0.1 increments (*k* = 1 corresponds to *k*I being equal to the diagonal of X’X). We use bootstrapping to construct confidence intervals for the parameters. The use of bootstrapping has the additional benefit of relaxing the assumption of normality of the parameter estimates. Therefore, it also tests the robustness of our results to violations of this assumption, which is anyway unlikely as with large samples like ours, the central limit theorem implies that the normal distribution is a good approximation of the distribution of the regression coefficients.

For both models, results indicate that the parameter estimates for the topography variables (volume, speed, min. required speed and circuitousness) remain identical for all values of *k* (to a 3-digit precision). Further, they remain significant at the same *p*-value as reported in Table 1, with the exception of speed which is significant at *p* < 0.05 in 6 cases, and at *p* < 0.10 in 4 cases.

**Ancillary Analyses**

To begin to explore a potential mechanism underlying the observed effect, we examined the link between the topography of thought and creativity.

To do so, we re-analyzed Studies 2, 3 and 6 data from Toubia and Netzer (2017). In each study, participants were asked to generate new ideas for a health-related smartphone app. Other participants then rated each idea based on how creative they thought it was (on a 5-point Likert scale). The ideas are rather short (M = 44.73 words), so we use windows of 5 words, and allow sentences to be split (otherwise we are left with too few ideas with at least 2 windows, which are required to compute speed and volume). Then, we examine the relationship between volume, speed, and judged creativity. We include fixed effects for each study, and following Toubia and Netzer (2017), use a random effects regression, to account for the fact that ideas are grouped by ideators (each participant typically generated multiple ideas). Given some ideas are longer than others, we also control for the number of words in the idea, the number of chunks in the idea (log-transformed), and the number of characters in the idea (log-transformed).

Results (Table S4) indicate that ideas that cover more ground are seen as more creative (*ß* = 0.066, *p* < 0.01, *z* = 3.78), as are ideas that do so with less speed (albeit marginally, *ß* = -0.028, *p =* 0.07, *z* = -1.80). Even including additional controls from Toubia and Netzer (2017), results remain the same (Model 2).

**Table S4**

*Topography of Thought and Creativity*

|  | Model 1 | Model 2 |
| --- | --- | --- |
| *Focal variables* |  |  |
| **Volume** | **0.066**** | **0.072**** |
| **Speed** | -0.028^ | -0.030^ |
| ***Additional controls from Toubia and Netzer (2017)*** |  |  |
| Distance to prototypical edge weight distribution | --- | -0.314** |
| Edge weight: average | --- | 1.297 |
| Edge weight: coeff. of var. | --- | 0.056 |
| Edge weight: min. | --- | 0.648* |
| Edge weight: max. | --- | -0.070 |
| Node frequency: average | --- | 1.062* |
| Node frequency: coeff. of var. | --- | 0.412 |
| Node frequency: min. | --- | 0.389 |
| Node frequency: max | --- | -0.474 |
| Size of semantic subnetwork | --- | -0.004 |
| ***Idea-specific controls*** |  |  |
| Log(# of characters) | yes | yes |
| Log(# of words) | yes | yes |
| Log(# of chunks) | yes | yes |
| ***Study fixed effects*** | yes | yes |
| ***Ideator random effects*** | yes | yes |
|  |  |  |
| Num parameters | 8 | 18 |
| Num observations | 1,294 | 1,294 |
| R^2^ | 0.276 | 0.299 |
| Adjusted R^2^ | 0.272 | 0.289 |

Note: All variables for which coefficients are reported are standardized. ^. p-value < .10 *: p-value < 0.05; **: p-value < 0.01.

**References**

Allen, M. P. (1997) “The problem of multicollinearity.” *Understanding regression analysis* 176-180.

Dormann, C. F., Elith, J., Bacher, S., Buchmann, C., Carl, G., Carré, G., ... & Lautenbach, S. (2013). Collinearity: a review of methods to deal with it and a simulation study evaluating their performance. *Ecography*, *36*(1), 27-46.

Toubia, O., Berger, J., & Eliashberg, J. (2021). How quantifying the shape of stories predicts their success. *Proceedings of the National Academy of Sciences, 118*(26), e2011695118.

Toubia, O., Netzer, O. (2017). Idea generation, creativity, and prototypicality. *Marketing Science, 36*(1), 1–20.
